# Comparison of UPDRS III score between young and late onset Parkinson disease after deep brain stimulation: A meta-analysis

**DOI:** 10.1097/MD.0000000000035861

**Published:** 2023-11-03

**Authors:** Jae Meen Lee, Kyoungjune Pak

**Affiliations:** a Department of Neurosurgery and Biomedical Research Institute, Pusan National University Hospital, Busan, Republic of Korea; b Department of Nuclear Medicine and Biomedical Research Institute, Pusan National University Hospital, Busan, Republic of Korea; c School of Medicine, Pusan National University, Busan, Republic of Korea.

**Keywords:** deep brain stimulation, Parkinson disease, subthalamic nucleus, Unified Parkinson’s Disease Rating Scale

## Abstract

**Purpose:**

Parkinson disease (PD) is a common age-related neurodegenerative disease. Subthalamic nucleus deep brain stimulation (DBS) is a safe and effective surgical treatment for medically resistant advanced PD. However, the relationship between the age at PD onset and the efficacy of subthalamic nucleus DBS surgery remains unclear. Thus, we conducted a meta-analysis to compare motor symptom improvements after DBS for the treatment of young-onset and late-onset PD.

**Methods:**

We systematically searched the Medline and Embase databases (from inception to March 2023) for English publications. All published studies comparing the outcomes (Unified Parkinson’s Disease Rating Scale part III [UPDRS III] scores) of DBS between the young-onset Parkinson disease (YOPD) and late-onset Parkinson disease (LOPD) groups were identified. The effect size was defined as the standardized mean difference (Hedge g) with 95% confidence intervals. The standardized mean difference was calculated by dividing the difference in UPDRS III scores between old and young patients by the pooled and weighted standard deviations. The meta-analysis was performed using R Statistical Software version 4.2.2 (The R Foundation for Statistical Computing).

**Results:**

Six studies were eligible for inclusion. The standardized mean difference of UPDRS III score between young and old patients ranged from −0.54 to 1.43 with a pooled difference of 0.0932 (95% confidence intervals: − 0.4666 to 0.6530, *I*^2^ = 86.77%). Subgroup analyses were performed with a cutoff age of 65 years and did not show a significant difference in UPDRS III scores between patients with YOPD and LOPD (0.1877, −0.6663 to 1.0417).

**Conclusions:**

The efficacy of DBS in patients with YOPD and LOPD showed similar improvements in the UPDRS score; hence, DBS should be considered, if necessary, regardless of the onset age of PD.

## 1. Introduction

Parkinson disease (PD) is one of the most common age-related neurodegenerative diseases.^[1]^ PD is well controlled by dopamine drugs, but their effectiveness gradually decreases due to long-term drug treatment and the side effects of these drugs. Subthalamic nucleus (STN) deep brain stimulation (DBS) is a safe and effective surgical treatment for medically resistant advanced PD with dyskinesia.^[2–4]^ STN DBS improves PD motor symptoms and drug-induced motor fluctuations in patients with advanced PD. The effects of DBS on PD have been reported in many studies.^[5–7]^ The degree of improvement in motor symptoms after DBS for PD was evaluated using the Unified Parkinson’s Disease Rating Scale part III (UPDRS III) scores.^[3,8–12]^

The relationship between the age at PD onset and the efficacy of STN DBS surgery remains unclear. Young-onset Parkinson disease (YOPD) is typically defined as symptom onset between the ages of 21 and 40 years.^[13–16]^ These symptoms are similar to those of late-onset Parkinson disease (LOPD) but are more severe and less responsive to medication. Therefore, it is difficult to predict the effectiveness of DBS in these 2 groups. Several studies have reported that DBS shows good results in both YOPD and LOPD.^[17–23]^ However, there are studies showing that DBS has a different effect on each group. Some studies have shown that DBS is more effective in improving the quality of life in YOPD than LOPD,^[24,25]^ whereas another study has reported that there is less acute improvement from acute DBS than LOPD.^[26]^

Hence, we divided previous publications into 2 groups according to the age of onset (young and old) and conducted this meta-analysis to compare motor symptom improvements in DBS for the treatment of YOPD and LOPD with advanced PD.

## 2. Materials and methods

### 2.1. Data search and study selection

We performed systematic searches of Embase/Medline (from inception to March 2023) for publications written in English using keywords such as “Parkinson disease” and “Deep brain stimulation.” All the searches were limited to human studies. All published studies comparing the outcomes (UPDRS III scores) of DBS between the YOPD and LOPD groups were identified. In order to objectively confirm the effectiveness of DBS, data comparing and analyzing scores of the “off” state before and after surgery were meta-analyzed. Patients were divided into 2 groups based on their age. Patients < 65 years of age were grouped in the young group and patients > 65 years of age were grouped in the old group. When performing treatment such as surgery, the majority of the elderly are based on the age of 65. Many studies have also divided the age group into 65 years, so we divided into groups at this age group.

Review articles, abstracts, editorials, and duplicate data were excluded. If more than one study was published by the same institution, only the report with the information most relevant to this study was included. Two authors performed the searches, screened the studies independently and resolved any discrepancies by reaching a consensus.

### 2.2. Data extraction and statistical analysis

Data were independently extracted from the publications by 2 reviewers, and the following information was recorded: first author, year of publication, country, number of young and old patients, and cutoff age. The effect size was defined as the standardized mean difference (Hedge g) with 95% confidence intervals (CI). The standardized mean difference was calculated by dividing the difference in UPDRS III scores between old and young patients by the pooled and weighted standard deviations. Heterogeneity among the studies was assessed using the *I*^2^ statistics.^[27]^ An *I*^2^ value >50% was considered indicative of substantial heterogeneity. When heterogeneity was observed, pooled analysis was performed based on the random-effects model; when heterogeneity was not observed, pooled analysis was performed based on the fixed-effect model. Statistical analyses were performed using R Statistical Software version 4.2.2 (The R Foundation for Statistical Computing).

## 3. Results

### 3.1. Study characteristics

**Figure 1.**
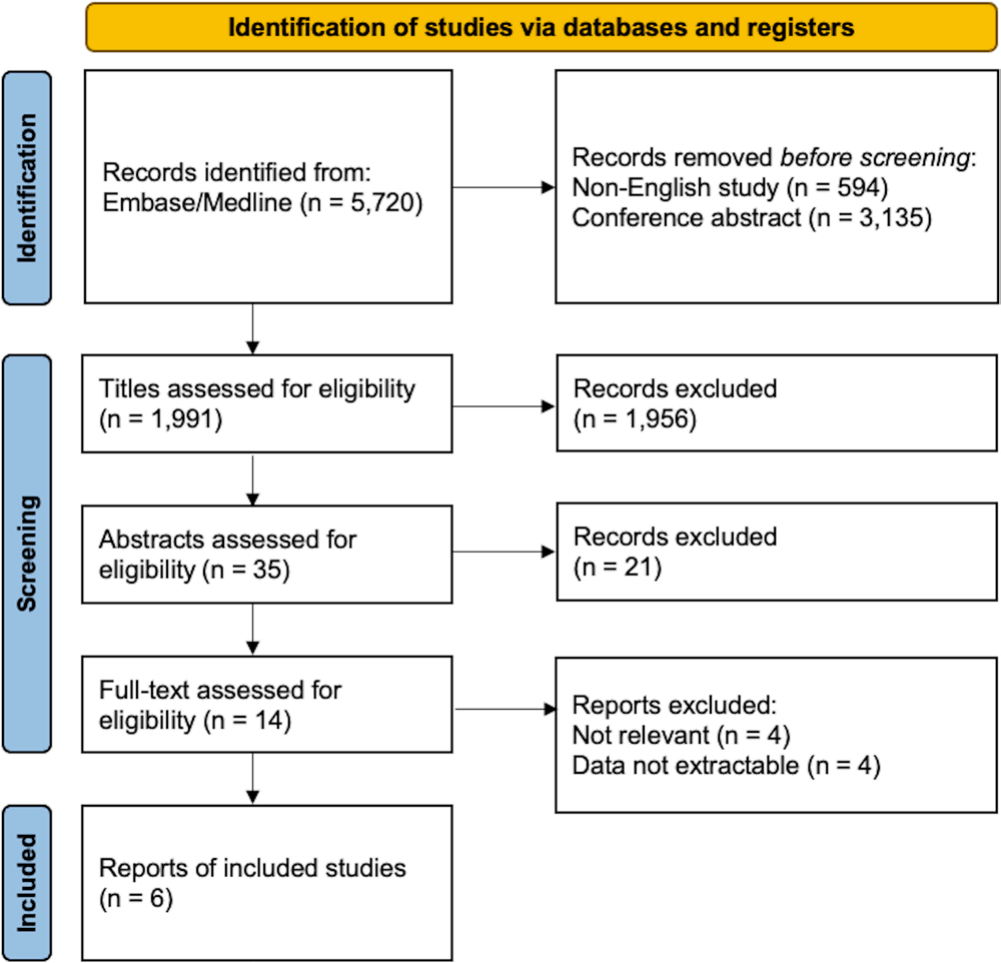
Flowchart for the inclusion of studies in this meta-analysis.

An electronic search identified 5720 articles. Conference abstracts (n = 3135), and non-English studies (n = 594) were excluded. Another 1977 studies that did not meet the inclusion criteria based on their titles and abstracts were excluded. After reviewing the full text of the remaining 14 articles, 6 studies were eligible for inclusion.^[17–19,24,25,28]^ Four studies divided the patients into 2 groups with an age cutoff of 65 years,^[17,19,24,28]^ one study with an age cutoff of 70 years,^[25]^ and one study with an age cutoff of 50 years.^[18]^ All patients were on medication with levodopa. The detailed procedure for inclusion is shown in Figure 1, and the study characteristics are summarized in Table 1.

**Table 1:** Studies included in the meta-analysis.

Author	Year of publication	Country	Age cutoff	No. of YOPD (M/F)	No. of LOPD (M/F)	Follow-up
Vats A	2019	United Kingdom	65	20 (13/7)	20 (15/5)	1-year, 2-year
Bouwyn JP	2016	France	65	57 (/24)	34 (/14)	6-month
Chiou SM	2016	Taiwan	70	56 (37/19)	16 (11/5)	6-month
Zhang J	2019	China	50	25 (14/11)	30 (14/16)	6-month, 1-year, 2-year, 3-year
Derost PP	2007	France	65	53 (38/15)	34 (24/10)	3-month, 6-month, 1-year, 2-year
Shalash A	2014	Germany	65	81 (51/30)	29 (18/11)	6-month~1-year, 3–5-year

F = female, LOPD = late onset Parkinson disease, M = male, YOPD = young onset Parkinson disease.

### 3.2. Comparison of UPDRS III score between young and old patients with Parkinson disease

**Figure 2.**
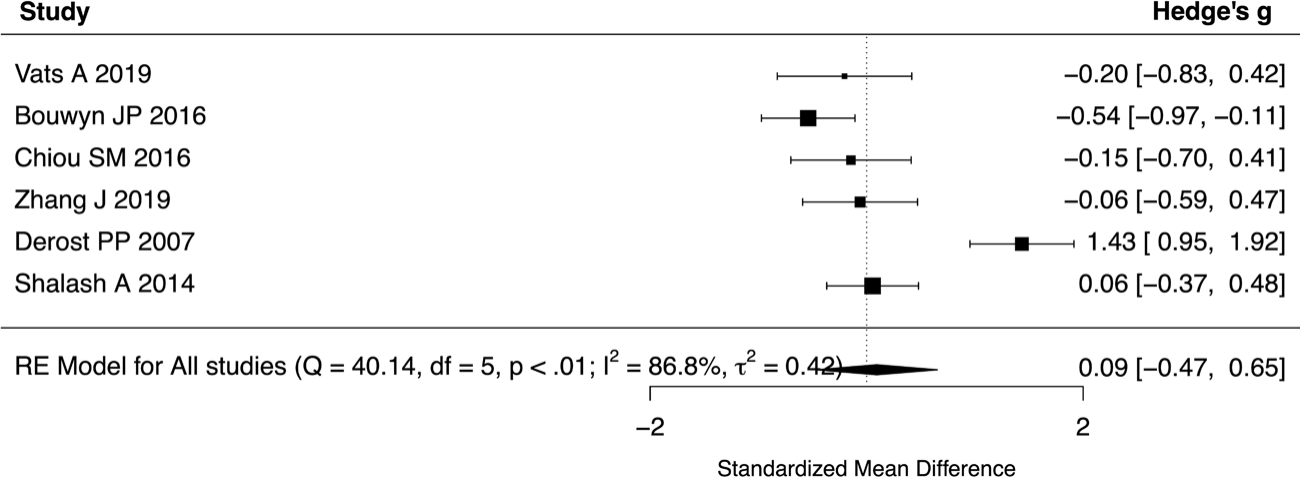
Forest plot comparing UPDRS III score between young and old patients at baseline. UPDRS III = Unified Parkinson’s Disease Rating Scale part III.

**Figure 3.**
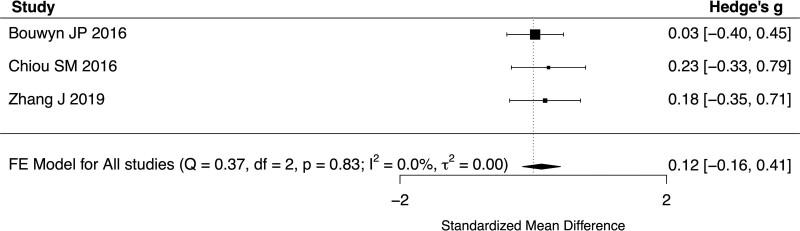
Forest plot comparing UPDRS III score between young and old patients at 6-month follow-up. UPDRS III = Unified Parkinson’s Disease Rating Scale part III.

**Figure 4.**
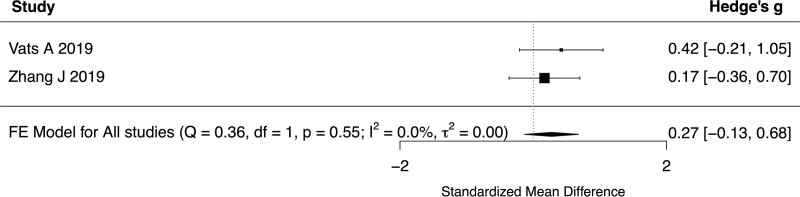
Forest plot comparing UPDRS III score between young and old patients at 1-year follow-up. UPDRS III = Unified Parkinson’s Disease Rating Scale part III.

**Figure 5.**
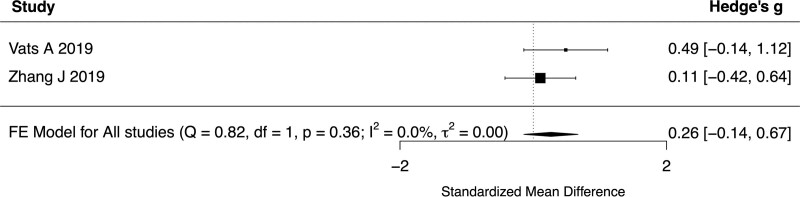
Forest plot comparing UPDRS III score between young and old patients at 2-year follow-up. UPDRS III = Unified Parkinson’s Disease Rating Scale part III.

All 6 studies reported baseline UPDRS III scores for both YOPD and LOPD. The standardized mean difference of UPDRS III score between young and old patients ranged from −0.54 to 1.43 with a pooled difference of 0.0932 (95% CI: −0.4666 to 0.6530, *P* = .7442, *I*^2^ = 86.77%, 6 studies, 455 patients) (Fig. 2). Subgroup analyses were performed with a cutoff age of 65 years and did not show a significant difference in UPDRS III scores between patients with YOPD or LOPD (SMD 0.1877, 95% CI −0.6663 to 1.0417, *P* = .6666, *I*^2^ = 92.09%, 4 studies, 328 patients). In addition, the UPDRS III score at 6-months (SMD 0.1234, 95% CI −0.1616 to 0.4085, *P* = .3961, *I*^2^ = 0%, 3 studies, 218 patients, Fig. 3), 1-year (SMD 0.2733, 95% CI −0.1321 to 0.6787, *P* = .1864, *I*^2^ = 0%, 2 studies, 95 patients, Fig. 4) and 2-years (SMD 0.2649, 95% CI −0.1409–0.6707, *P* = .2008, *I*^2^ = 0%, 2 studies, 95 patients, Fig. 5) of follow-up did not show a significant difference between YOPD and LOPD.

## 4. Discussion

PD is a neurodegenerative disease characterized by various clinical symptoms including tremors and bradykinesia.^[1]^ This occurs due to dopamine deficiency in the basal ganglia; dopamine-based drug treatment is very effective, of which levodopa is a representative example. These drugs are very effective for patients with PD; therefore, symptoms can be well controlled using medication only. However, as the treatment period increases, the drug effectiveness decreases by “wearing off” due to the side effects of levodopa.^[29,30]^ When these symptoms occur, it becomes difficult to expect improvement, even if the dose of the drug is increased. In this case, DBS was the representative consideration for surgical treatment. Among these, STN DBS has been performed the most, and many studies have reported that it is very effective in advanced PD.^[5–7,31]^ Usually, the STN is easily identified by MR T2 imaging, and several studies have been a number of researches that investigated symptom improvements according to different regions of the STN. Regarding PD, several studies have found that maximal stimulation benefits are achieved when the electrodes are located at the dorsolateral STN border.^[32]^ This intraoperative evaluation *via* stimulation-induced improvement allowed us to confirm the most effective electrode locations.

Age and the degree of preoperative drug effects are known to affect the postoperative results.^[33]^ Similar to other surgeries, age is an important determinant of DBS. Most PD cases occur in old age, but they can also occur at an early age; and are called YOPD. YOPD and LOPD are similar,^[34,35]^ but are characterized by differences in clinical characteristics, disease progression, and levodopa response.^[36–39]^ Although patients in the YOPD group did not differ significantly from those in the LOPD group with respect to disease severity or disability, dyskinesia was more frequent and severe in the YOPD group.^[38,40,41]^ YOPD patients develop dyskinesia earlier than LOPD patients, and it is difficult to manage them with medications.^[14,42]^ In addition, YOPD progresses more slowly than LOPD.^[16,43]^ On the other hand, the severity of dyskinesia is greater in LOPD patients than YOPD patients. Patients with LOPD suffer from greater motor impairment than those with middle-aged Parkinson disease, and this difference may be due to faster disease progression and less effective medical treatment.^[44]^ Consequently, both require surgical treatment such as DBS at an appropriate time.

Several studies have reported that patients with YOPD and LOPD demonstrate reductions in UPDRS III scores.^[17–23,45]^ Vesper et al^[46]^ showed that there is no significant difference in the quality of life after STN DBS between young and old patients with PD during follow-up or improvement in motor symptoms. This was a result of little improvement in cognitive function and was not caused by differences in motor symptom improvement after DBS.^[24]^ Several studies have reported that LOPD is associated with a high risk of complications of DBS.^[28,46,47]^ Therefore, there is a tendency to avoid DBS for LOPD. Conversely, postoperative motor complications have been reported to occur more frequently and severely in YOPD.^[38]^ Since the risk of postoperative complications was accompanied by risks in both groups, it should be emphasized that there was no difference between the 2 groups in improving motor symptoms.

Our study included 6 publications with 455 (292 vs 163) patients and reflected the latest effects of DBS for the treatment of PD according to onset age. This meta-analysis aimed to compare the impact of DBS on motor symptoms in the treatment of PD between late and early onset patients.

Vats et al^[19]^ and Bouwyn et al^[28]^ added a comparison of changes in cognitive function, Derost et al^[24]^ compared changes in quality of life, and Zhang et al^[18]^ and Chiou et al^[25]^ added a comparative analysis of reduction in daily levodopa equivalent dose. Shalash et al^[17]^ showed that the duration of the disease does not differ in UPDRS III score, but there is a difference in dyskinesia. Our meta-analysis showed that the effects were similar by comparing the UPDRS III score of the 2 groups, but it seems necessary to analyze several other items compared in other studies in the future to produce better results. This meta-analysis demonstrated the motor benefits of STN DBS in both patients with YOPD and those with LOPD. This suggests that DBS is also effective for LOPD, and when age is taken into consideration, patients with LOPD should not preclude DBS treatment because of the high risk.

This study had several limitations. First, only a small number of studies were included in this meta-analysis. After a systematic search of the publications, several studies were assessed for eligibility. However, most were published by the same institution with overlapping subjects, and we selected the most relevant publications from the pool of articles with the same subjects. Secondly, the focus was only on improving motor symptoms using the UPDRS score. Other PD symptoms, such as cognition and balance, were not considered during DBS surgery. Although further work is needed to investigate the potential impact of the abovementioned limitations on the DBS effect between patients with YOPD and LOPD, our study provides clinically meaningful insights into the possible role of age in DBS. Therefore, caution should be exercised when interpreting the findings of studies included in this meta-analysis. In addition, we could not extract the change of UPDRS III score between baseline and follow-up from the studies included in this meta-analysis. Therefore, further studies are needed to investigate the comparison of the effectiveness of DBS surgery between 2 groups.

In conclusion, the efficacy of DBS for patients with YOPD and LOPD showed similar improvements in the UPDRS score; hence, DBS should be considered, if necessary, regardless of the onset age of PD. Further prospective studies involving postoperative care under the same conditions are needed to elucidate the differences in the effects of DBS between the YOPD and LOPD groups.

## Author contributions

**Conceptualization:** Kyoungjune Pak.

**Data curation:** Jae Meen Lee, Kyoungjune Pak.

**Formal analysis:** Jae Meen Lee.

**Project administration:** Kyoungjune Pak.

**Software:** Kyoungjune Pak.

**Writing – original draft:** Jae Meen Lee.

**Writing – review & editing:** Jae Meen Lee, Kyoungjune Pak.
